# *luxS* Mutant Regulation: Quorum Sensing Impairment or Methylation Disorder?

**DOI:** 10.3390/s120506176

**Published:** 2012-05-10

**Authors:** Qian Wang, Zhiyan He, Yuejian Hu, Yuntao Jiang, Rui Ma, Zisheng Tang, Jingping Liang, Zheng Liu, Zhengwei Huang

**Affiliations:** 1 Department of Endodontics, Ninth People's Hospital, Shanghai Key Laboratory of Stomatology, School of Medicine, Shanghai Jiao Tong University, No. 639 Zhizaoju Road, Shanghai 200011, China; E-Mails: qtdc1010@sjtu.edu.cn (Q.W.); zyhe23@126.com (Z.H.); hyjian@sjtu.edu.cn (Y.H.); jiangyuntao1974@163.com (Y.J.); marui6868@yahoo.com (R.M.); tangzisheng@yahoo.com.cn (Z.T.); sris@sh163.net (Z.L.); 2 Department of Endodontics, The Affiliated Stomatology Hospital of Tongji University, School of Stomatology, Tongji University, Shanghai 200072, China

**Keywords:** quorum sensing, AI-2, *luxS*, *sahH*

## Abstract

AI-2–mediated quorum sensing has been identified in various bacteria, including both Gram-negative and Gram-positive species, and numerous phenotypes have been reported to be regulated by this mechanism, using the *luxS*-mutant strain. But the AI-2 production process confused this regulatory function; some considered this regulation as the result of a metabolic change, which refers to an important metabolic cycle named activated methyl cycle (AMC), caused by *luxS*-mutant simultaneously with the defect of AI-2. Herein we hypothesized that the quorum sensing system—not the metabolic aspect—is responsible for such a regulatory function. In this study, we constructed plasmids infused with *sahH* and induced protein expression in the *luxS*-mutant strain to make the quorum-sensing system and metabolic system independent. The biofilm-related genes were investigated by real-time polymerase chain reaction (PCR), and the results demonstrated that the quorum-sensing completed strain restored the gene expression of the defective strain, but the metabolically completed one did not. This evidence supported our hypothesis that the autoinducer-2-mediated, quorum-sensing system, not the AMC, was responsible for *luxS* mutant regulation.

## 1. Introduction

Quorum sensing (QS) is an intercellular signal mechanism that bacteria use to control their gene expression for adapting to changes in their surroundings. This process involves the production, secretion, and recognition of signal molecules and regulation of gene expression [1]. Among these signal molecules, autoinducer-2 (AI-2), which was first identified as a regulator of bioluminescence in *Vibrio harveyi* [2], supposedly participates in interspecies cell-to-cell communication because of its generating gene *luxS* that conservatively exists in a broad range of species [3].

**Figure 1.**
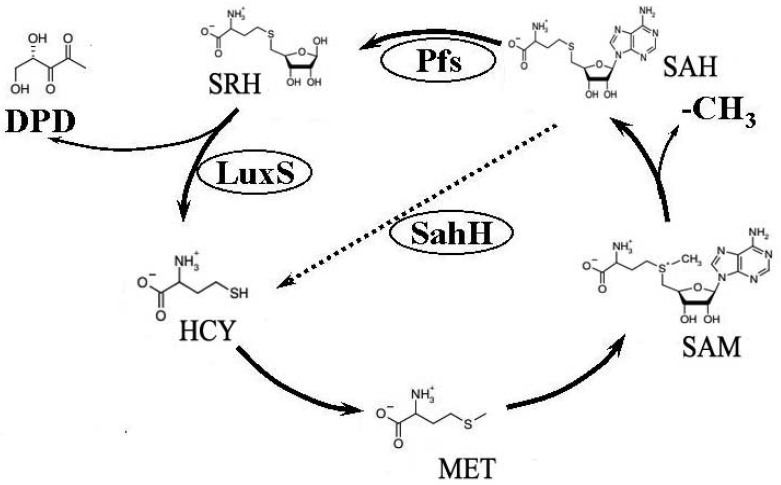
Process of activated methyl cycle (AMC; adapted from Vendeville *et al.* [18]). *E. coli* uses a two-step mechanism involving the Pfs and LuxS enzymes to produce the AI-2 precursor and homocysteine (HCY), while *P. aeruginosa* synthesizes HCY from S-adenosylhomocysteine (SAH) in a one-step reaction involving the SahH enzyme.

Previous studies have used the *luxS* mutant strain to demonstrate that AI-2–mediated QS plays an important regulative role in many biological behaviours of bacteria such as cell division, DNA processing, virulence, and motility [4–8]. Furthermore, as an important etiological factor of infectious diseases [9–11], biofilm formation also differs between *luxS* mutant and its wild-type strain [12–15]. There is much controversy over the origin of these regulating behaviors. AI-2 is a byproduct of the activated methyl cycle (AMC) [16] (Figure 1). This important metabolic cycle begins with homocysteine (HCY), then involves the formation of methione (MET), *S*-adenosylmethionine (SAM), and *S*-adenosylhomocysteine (SAH). During the cycle, SAM provides an activated methyl group (CH_3_), which is used for the methylation of RNA, DNA, certain metabolites, and proteins [17,18]. To complete the cycle, HCY is generated from SAH through two independent pathways. Some bacteria, like *Escherichia coli*, travel one pathway that needs Pfs enzyme to first produce *S*-ribosylhomocysteine (SRH), then LuxS catalyzes the conversion from SRH to HCY, simultaneously generating the precursor of AI-2, 4,5-dihydroxy-2,3-pentanedione (DPD). The other route involves a one-step conversion catalyzed by SAH hydrolase (SahH), which catalyzes the conversion from SAH to adenosine and HCY but without generating DPDs [19–21], bacteria such as *P. aeruginosa* which do not contain *luxS* passing through this way. Since most previous studies on mutated *luxS* considered it a QS-defective strain and that AMC was also disrupted at the LuxS level, a series change would occur in the bacteria's metabolism [20]. Thus arose the controversy as to whether the change in gene expression is caused by QS, as previously described, or by AMC metabolic pathway disruption [21,22].

Although the *luxS* mutant leads to AMC obstruction, it is not believed to arouse so much regulated gene expression belonging to such diverse biological behaviors as gene production, virulence, and motility [7,23]. Such a wide influence is likely to be due to a monolithic mechanism, such as AI-2-induced QS. Furthermore, in previous studies, mutated *luxS* was not fatal to the strain, so we speculated that the influential range of the AMC obstacle was limited. To this end, we hypothesized that the QS role, instead of an AMC obstacle, is responsible for *luxS* mutant regulation. To verify our hypothesis, we intended to express SahH in an *E. coli luxS* mutant strain, aiming to repair the AMC of this strain but retain the defective QS system, then detect whether the genes reported to be regulated by AI-2–mediated QS would be restored.

## 2. Materials and Methods

### 2.1. Bacterial Strains, Plasmid Construction, and Culture Condition

The strains and plasmids used in this study are listed in Table 1, the primers in Table 2. The *sahH* gene was amplified from genomic DNA of the *P. aeruginosa* strain PAO1 using primers SF and SR, and the *luxS* gene was amplified from genomic DNA of *E. coli* W3110 with primers LF and LR. The restriction enzymes chosen in this part were BamH I and EcoR I for *sahH* and vector, EcoR I and Bgl II for *luxS*. And Bgl II is the isocaudamer of BamH I. To construct the plasmids p*luxS* and p*sahH*, amplified products were inserted into the expression vector pGEX4T-1, using the standard method of preparing clones [24]. Generally, DNA amplification products were digested by the corresponding restriction enzymes and ligated with the vector. After sequencing, p*luxS*, p*sahH*, and pGEX4T-1 were transformed into strain MDAI2 (*luxS* mutant strain) to construct strains M-L, M-S, and M-P, respectively.

All strains were precultured in a 2 × YT medium at 37 °C overnight with shaking at 220 rpm, and the concentration of ampicillin for selection was 100 μg/mL. These overnight cultures were inoculated into a fresh medium and cultivated at 37 °C with 250-rpm shaking. When the strains had reached the exponential phase, a final concentration of 0.1 mmol/L isopropyl-*β*-D-thiogalactopyranoside (IPTG) was added, and cultivation was continued for about 6 h.

### 2.2. Western Blotting

After cultivation, the OD_600_ of five strains was tested and total proteins were extracted. Briefly, about 2 × 10^8^ *E. coli* were collected and a corresponding volume of double-distilled water (ddH_2_O) and a 5× sample of buffer (20% sodium dodecyl sulfate [SDS], 20% glycerol, 200 mM Tris base, pH 6.8, 0.001% bromophenol blue) was used to subsequently suspend the pellets. The samples were heated at 100 °C for 10 min and centrifuged for 1 min. Equal amounts of total proteins were electrophoresed in SDS–10% polyacrylamide gel electrophoresis. Western blotting was performed as previously described [24]. The antibodies used in this study were monoclonal anti-glutathione-S-transferase (anti-GST) and rabbit antimouse antibody with a green fluorescent group.

### 2.3. RNA Extraction and Reverse Transcription

After IPTG induction and cultivation for 6 h, the total RNA of these strains was extracted. Culture volumes equivalent to 10 mL with an OD_600_ of 1.0 were harvested by centrifugation. The pellet was suspended with TRIzol (Invitrogen, Carlsbad, CA, USA). The reagent and RNA extraction process followed the manufacturer's specifications. The resulting RNA was dissolved in diethyl pyrocarbonate-treated water and stored at −80 °C. The PrimerScript gDNA eraser RT reagent kit (Takara, Otsu, Shiga, Japan) was used to generate cDNA; about 1 μg of total RNA was used for each strain. To detect the target exogenous gene, cDNA of each strain was used as a template for polymerase chain reaction (PCR). Other compositions were forward/reverse primer (SF, SR, LF, LR), PrimeStar HS DNA Polymerase (Takara) and buffer, dNTP mixture, and ddH_2_O. PCR conditions included an initial denaturation at 98 °C for 5 min, followed by 30 cycles amplification consisting of 98 °C for 15 s, 55 °C for 15 s, and 72 °C for 90 s, then final extension at 72 °C for 5 min. The amplified products were electrophoresed in 1% agarose gel.

### 2.4. Real-Time PCR

To determinae whether the AMC-completed strain would restore the biofilm formation-related genes affected by *luxS* mutant, the expression of these genes was compared with the real-time PCR. The target genes and their primers are shown in Table 2. The amplification efficiency and template specificity of each primer pair were verified, then the amplification was performed with the following 15-μL reaction mixture: 7.5 μL 2× Thunderbird SYBRqPCR Mix (Toyobo, Osaka, Japan), 5 μL cDNA template, 1 μL PCR primers mix (10 μM), and 1.5 μL ddH_2_O. PCR conditions included an initial denaturation at 98 °C for 5 min, followed by 40 cycles amplification of 98 °C for 15 s, 55 °C for 15 s, and 72 °C for 30 s. To check DNA contamination, the production of RT-PCR step 1 (without reverse transcriptase) served as a negative control. The *rpoA* gene was used in this study as the normalising gene for all reactions [26]. Applied Biosystems (Carlsbad, CA, USA) 7900HT Fast Real Time PCR System was used for this test, and fold changes of the expression levels were calculated by SDS Software v2.3 with RQ Study 1.2 (Applied Biosystems). All the assays were conducted with each sample in triplicate. Here, the Kruskal-Wallis test was used to analyse the gene expression difference among the five strains. A *P*-value less than 0.05 was considered statistically significant.

## 3. Results and Discussion

### 3.1. Identification of Exogenous Gene Expression

To test and verify our hypothesis, the QS system and AMC must be independent. Previous investigators have collected supernatant that had cultivated a wild-type strain for some time, believing it to contain AI-2 [27,28]. They then used it as a conditioning medium in which to cultivate the *luxS* mutant strain. In a sense, this method could make the QS system and AMC independent, but it allowed the other substance in the supernatant—secreted by the bacteria—to influence the results. This study, aiming to restore the obstructed AMC and simultaneously keep the defective QS mechanism, transformed the *sahH* into an *E. coli luxS* mutant strain. Consequently, we could now check whether the AMC or the QS system took responsibility for changes caused by the *luxS* mutant.

**Figure 2.**
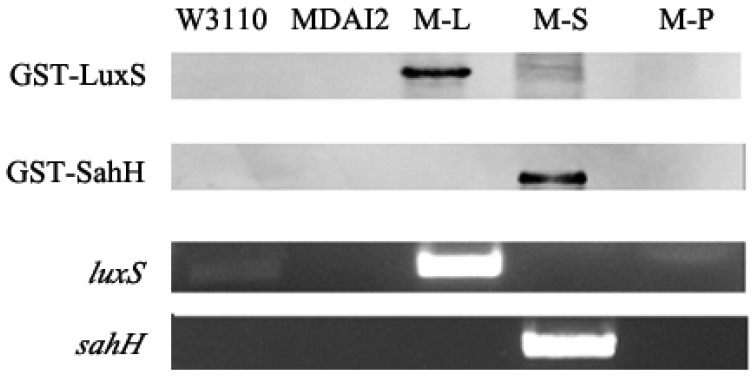
GST-tagged LuxS and SahH were detected in strains M-L and M-S, respectively, by Western blotting. RT-PCR showed that *luxS* existed in both W3110 and M-L, while M-L is more likely caused by gene overexpression. *sahH* was detectable only in M-S.

The RT-PCR and Western blot were performed to prove the expression of target genes in these five strains. As shown in Figure 2, the bands detectable by anti-GST represented the fusion proteins GST-LuxS and GST-SahH, whose molecular weights were about 44.9 kDa and 77.7 kDa, respectively. For *luxS* RT-PCR, the primer pairs LF and LR could spot both W3110 and M-L, whereas *sahH* was detected only in M-S. Moreover, the band amplified from M-L is more remarkable than W3110, which amplification should be attributed to the overexpression of p*luxS*.

### 3.2. Gene Expression Differences Caused by luxS Mutant are Regulated by the QS System, not by AMC

To determine whether AMC regulates gene expression of the *luxS* mutant strain, a real-time PCR was conducted. Biofilm formation is one of the main causative factors in infectious diseases. And as described before, biofilm formation was reported to be regulated by quorum sensing system. Genes such as *fliA*, *fliC*, *motA*, and *motB*, which were responsible for flagellar biosynthesis and rotation, also played an important role in the initial stage of the biofilm formation process [29–31]. They were also reported to have different expression levels between the wild-type and *luxS* mutant strain [14,15], so here these four genes were tested [32–34].

**Figure 3.**
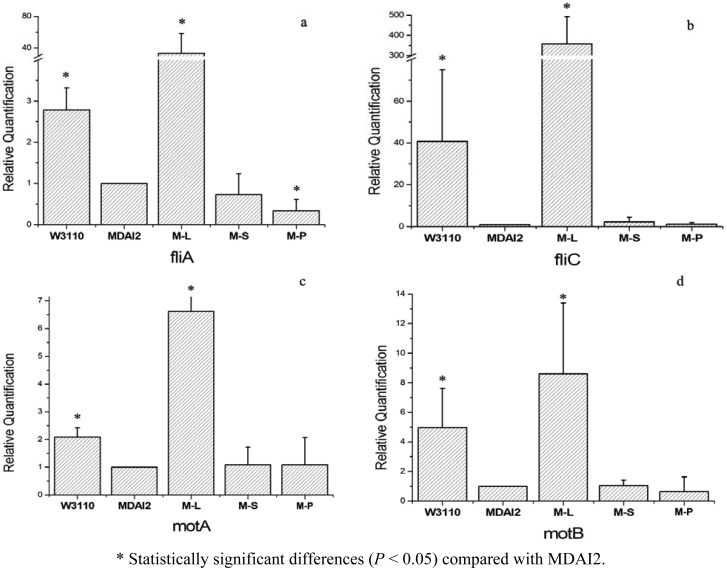
Real-time PCR was used to demonstrate that motility-related genes are restored in the *luxS*-mutant strain by the expression of *luxS*, although it was not restored in the mutant strain with the plasmid-expressing *sahH*. The results represent the means and standard deviations (SDs) of relative quantification.

The results of real-time PCR are shown in Figure 3. In general, M-S, relative to M-L, could not restore the gene expression of the *luxS* mutant strain. In practical terms, all the detected genes showed the trend that the wild-type strain expressed more than the *luxS* defective strain. They increased about 2.8-fold, 40.8-fold, 2.1-fold, and 5.0-fold for fliA, fliC, motA, and motB, respectively. But M-S, whose AMC disorder may be partly repaired, meanwhile the AI-2 producing process obstructed, had an expression level similar to the *luxS* mutant strain, MDAI2, and their relative expression folds were 0.73 for fliA, 2.3 for fliC, and 1.1 for motA and motB. That means SahH failed to restore the changes caused by *luxS* mutant. Under the same experimental conditions, the expression of target genes in the M-L strain was recovered or even up-regulated compared with the wild-type strain. These results showed that there was no significant restoration by expression of SahH which was intended to complete the AMC, thus the *luxS*-mediated QS system impacted the biofilm-related gene expression.

This study also showed that, the genes, downregulated by the *luxS* mutant, were even strongly upregulated when LuxS was overexpressed. This demonstrated that the mutant strain with LuxS expression may complete the QS system and cause AI-2 secretion to recover the expression of the target genes. And theoretically, overexpressed LuxS synthetized more active AI-2, so these target genes were upregultaed much more than the wild-type strain. But under the same experimental conditions, the mutant strain with SahH expression, which might recover the AMC system without AI-2 secretion, kept the expression of target genes at a low level (Figure 3), which means that, without integrated QS mediating, the phenotype could not be recovered, although its role in metabolism may be intact.

## 4. Conclusions

The genetic test results confirm our hypothesis that the QS role—rather than metabolism—is the primary regulator of *luxS* mutant physiological changes. We intend, in a future study, to further verify our hypothesis by means of an *E.coli* function test.

## Figures and Tables

**Table 1.** Plasmids and strains used in this study.

**Strain or plasmid Plasmids**	**Relevant genotype**	**Reference or source**
pGEX4T-1	expression vector, Ap^r^	Shanghai key laboratory of stomatology
p*luxS*	*luxS* from *E. coli* W3110 cloned in pGEX4T-1	this study
p*sahH*	*sahH* from *P. aeruginosa* PAO1 cloned in pGEX4T-1	this study
Strains		
*E.coli*		
W3110	K-12 strain, wild type	[25]
MDAI2	W3110 *luxS*∷TcrW3110-derived *luxS* mutant strain	[25]
M-L	MDAI2 p*luxS*	this study
M-S	MDAI2 p*sahH*	this study
M-P	MDAI2 pGEX4T-1	this study
*P. aeruginosa*		
PAO1	wild type	Shanghai key laboratory of stomatology

**Table 2.** Primers used in this study.

**Primer**	**Sequence (5′-3′)**
SF	GGCGGATCCATGAGCGCTGTCATGACGC *
SR	GGCGAATTCTTAGTAGCGATAGGTGTCCGG
LF	GGCAGATCTATGCCGTTGTTAGATAGCTTCAC
LR	GGCGAATTCCTAGATGTGCAGTTCCTGCAACT
fliAF	CCGCAACGCCACGGAAACTGA
fliAR	GCTCTTCGCGCCACTCATCGTA
fliCF	ATTGCTAACCGTTTCACCTCTAA
fliCR	CGCTGTAAGTTGTTGTTGATTTCG
motAF	CGTCGCTCCAAATACACCAA
motAR	CAGCGAAAACATCCCCATCT
motBF	GCCAGCGGTGAGAAAGGA
motBR	CAACCCTCCGACCATCAGTT
rpoAF	GCGCTCATCTTCTTCCGAAT
rpoAR	CGCGGTCGTGGTTATGTG

* Underline sequence is reference the restriction enzymes.
